# Performance Evaluation and Validation of QCM (Query Control Mechanism) for QoS-Enabled Layered-Based Clustering for Reactive Flooding in the Internet of Things

**DOI:** 10.3390/s20010283

**Published:** 2020-01-03

**Authors:** Fawad Ali Khan, Rafidah Md Noor, Miss Laiha Mat Kiah, Ismail Ahmedy, Idris Mohd Yamani Idna, Tey Kok Soon, Muneer Ahmad

**Affiliations:** 1 Department of Computer System & Technology, Faculty of Computer Science & Information Technology, University Malaya, Kuala Lumpur 50603, Malaysia; misslaiha@um.edu.my (M.L.M.K.); ismailahmedy@um.edu.my (I.A.); yamani@um.edu.my (M.Y.I.I.); koksoon@um.edu.my (T.K.S.); 2 Centre for Mobile Cloud Computing Research (C4MCCR), Faculty of Computer Science and Information Technology, University of Malaya, Kuala Lumpur 50603, Malaysia; 3 Department of Information System, Faculty of Computer Science & Information Technology, University Malaya, Kuala Lumpur 50603, Malaysia; mmalik@um.edu.my

**Keywords:** QoS, redundant query, Internet of things, network flooding, energy efficiency

## Abstract

Internet of Things (IoT) facilitates a wide range of applications through sensor-based connected devices that require bandwidth and other network resources. Enhancement of efficient utilization of a heterogeneous IoT network is an open optimization problem that is mostly suffered by network flooding. Redundant, unwanted, and flooded queries are major causes of inefficient utilization of resources. Several query control mechanisms in the literature claimed to cater to the issues related to bandwidth, cost, and Quality of Service (QoS). This research article presented a statistical performance evaluation of different query control mechanisms that addressed minimization of energy consumption, energy cost and network flooding. Specifically, it evaluated the performance measure of Query Control Mechanism (QCM) for QoS-enabled layered-based clustering for reactive flooding in the Internet of Things. By statistical means, this study inferred the significant achievement of the QCM algorithm that outperformed the prevailing algorithms, i.e., Divide-and-Conquer (DnC), Service Level Agreements (SLA), and Hybrid Energy-aware Clustering Protocol for IoT (Hy-IoT) for identification and elimination of redundant flooding queries. The inferential analysis for performance evaluation of algorithms was measured in terms of three scenarios, i.e., energy consumption, delays and throughput with different intervals of traffic, malicious mote and malicious mote with realistic condition. It is evident from the results that the QCM algorithm outperforms the existing algorithms and the statistical probability value “P” < 0.05 indicates the performance of QCM is significant at the 95% confidence interval. Hence, it could be inferred from findings that the performance of the QCM algorithm was substantial as compared to that of other algorithms.

## 1. Introduction

The Internet of Things (IoT) has become quite famous in the recent years in that many of our daily routine devices are being connected with us, covering many capabilities such as sensing, autonomy, and contextual awareness [1]. The IoT, resulting from Internet progress and the innovative evolution of smart devices, has led to the development of new computing prototypes. IoT is the next revolutionary technology that converts the present communication infrastructure into a completely futuristic network [2]. IoT is expected to contain high numbers of sensors collecting and passing on data on environmental conditions, physiological measurements, machine operational data, etc. IoT provides an integration of various sensors and objects that could communicate directly with one another without human intervention [3,4]. The primary purpose of the IoT is to allow secure data exchange between the real world devices and applications [5,6,7].

IoT promises a smart environment that would save time, energy, good quality of service (QoS), resources, and there will be less delay as compared to traditional wireless sensor networks [8]. Dynamic resource scheduling for heterogeneous workloads in IoT is critical to ensure QoS, level of energy consumptions on each mote, and traffic delay during data transmission [9]. Energy consumptions, QoS, and delay are the major challenging requirements for IoT networks, since data transmission in IoT network is based on priority [10,11,12,13,14,15,16]. Reference [17] proposed an adaptive meta-heuristic search for redundancy in IoT networks using the AntClust technique. Reference [18] used process-querying techniques to develop an enabling business intelligence for resource-constrained devices. Reference [19] proposed a scalability mechanism for IoT devices. Since scalability has become an important aspect that needs to be considered in any IoT system, the proposed mechanism enables IoT devices to be adaptable to environmental change. In addition, a three-level framework for IoT redundancy control was proposed by [20]. Reference [21] used a divide-and-conquer (DnC) method to develop an approach for improving energy efficiency in QoS-constrained WSNs (wireless sensor networks). Reference [22] proposed a node-level energy efficiency protocol for IoT devices to improve the energy efficiency in an IoT network. Reference [23] proposed a QoS architecture for IoT and cloud computing platforms to enable public/users to have easy access over diversified smart applications and services, distributed in the cloud with one IoT-enabled Intelligent Smart Card (ISC), through mobile devices with assured quality of service. In addition, modeling QoS in IoT applications was proposed by [24]. Reference [25] discussed network architecture and QoS issues in the IoT for a smart city. Reference [26] proposed a discrete component circuit implementation model together with its computational simulations using Bouali’s system.

**Figure 1:**
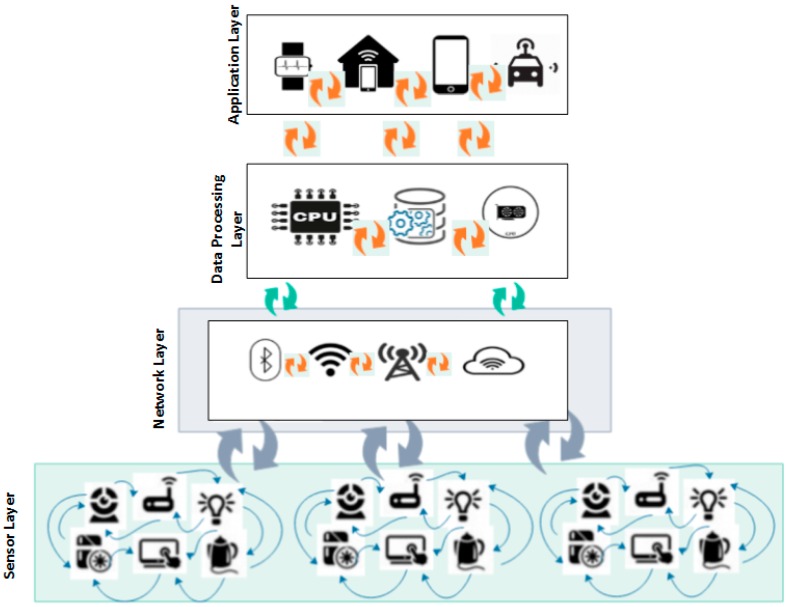
Layered-based system model with different motes communicating redundantly in IoT.

The primary purpose of the IoT is to allow secure data exchange between the real world devices and applications [27,28,29]. It is a known fact that IoT has the potential for a wide range of applications relating to agriculture, transportation, health, education, supply chain, farming, plant disease diagnosis, poultry, irrigation, and pest control [30,31]. Each application requires many sensors to connect and communicate with another, which may reduce the QoS of the network due to inefficient resource utilization, traffic delay due to redundant messages/queries because each device has direct access to cloud resources and energy consumption [32,33]. Layered-based system model with different motes communicating redundantly in IoT is illustrated in Figure 1 [34].

IoT is therefore based upon the integration of several communication solutions, identification and tracking technologies, sensor and actuator networks and distributed smart objects [33]. These objects/devices are connected to each other and share the same network for communicating with each other. All the devices are connected with the sensor to detect the particular surrounding conditions and analyze the situation and work accordingly. IoT devices are also programmed to take a decision automatically [34], according to the user so that the user can make the best decision. This interconnected network can bring lot of advancement in the technology of application and services that can bring economic benefit to the global business development. Many devices are connected to the internet to share local information in cyberspace [35,36,37].

Figure 1 shows the system model with different sensor motes communicating with each other between the physical (sensor) and network layers of IoT. From the figure, it can be seen that redundant messages, unwanted queries and network flooding are the major causes of inefficient utilization of resources, thus resulting in IoT devices consuming more energy with a high computational time (i.e., delay in data transmission), which in turn affects the network QoS [4]. Moreover, solving these issues in IoT networks is demanding due to the constraint nature of the devices with limited energy. Presently, to the best of our knowledge, no mechanisms for identification of redundant queries have been developed in this domain.

We examine several approaches for tackling unwanted and redundant communication in IoT networks to enable us to understand the sequence of actions that take place when flooding happens and propose a Cluster-Based Flooding (CBF) technique. The proposed technique is an interoperable solution both for physical layer and network layer devices. CBF divides the network into different clusters; local queries information are proactively maintained by the Intralayer clustering (IALC), while Interlayer clustering (IELC) is responsible for reactively obtaining the routing queries to the destinations outside the cluster. CBF is a hybrid approach, having the potential to be more efficient than traditional schemes in term of query traffic generation.

**Figure 2:**
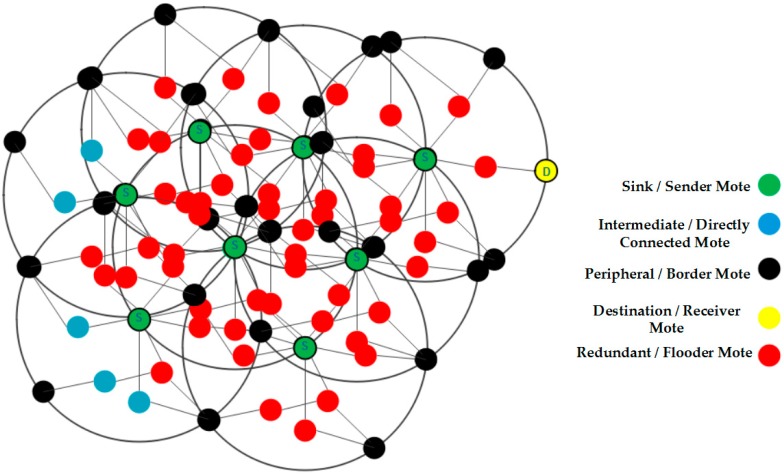
Flooding as a result of redundant queries.

A QoS-enabled QCM model is developed, and the results of the simulation show the superior performance against state of the art approaches in terms of traffic delay, QoS throughput, and energy consumption, under various performance metrics compared with traditional flooding and state of the art. In order to figure out real understanding of flooding in IoT networks, we provide modeling of the redundant queries which leads to flooding in Figure 2 [34].

## 2. Motivation

The Internet of Things has gained substantial attention over the last few years because of connecting daily things in a wide range of application and domains. Many sensors require bandwidth and network resources to give-and-take queries among heterogeneous IoT networks. The network sometimes becomes unable to handle unwanted and redundant queries generated from different smart devices. In addition, the network is also not able to prioritize the important queries among flooded queries. This research developed a new query control mechanism that could manage priority queries and refrain redundant and unwanted queries. This idea was able to save time and resources of networks with an efficient query management. Further, the performance evaluation of such query control mechanisms required inferential analysis of simulated results to statistically validate the performance parameters of QCMs under discussion.

## 3. Problem Statement

Network flooding is a key questioning strategy for successful exchange of queries. However, the risk of the original flooding is prone to unwanted and redundant network queries which may lead to cause heavy network traffic. Redundant, unwanted and flooded queries are the major cause of inefficient utilization of resources. IoT devices consume more energy and high computational time as compare to wireless sensor networks [15]. More queries lead to consumption of bandwidth, increase cost, and degrade QoS. Current existing approaches focus primarily on how to speed up the basic routing for IoT devices. However, solutions for flooding are not being addressed. This research proposed a new query control mechanism and evaluated its performance by statistical means.

## 4. Methodology

This research is based on the hypothesis that the proposed **QCM (Query Control Mechanism)** algorithm (Khan, F. A., Noor, R. M., Mat Kiah, M. L., Noor, N. M., Altowaijri, S. M., & Rahman, A. U., 2019) outperforms the other existing algorithms, i.e., DnC, SLA, and Hy-IoT for QoS-enabled layered-based clustering for reactive flooding in the Internet of Things. Table 1 presents the important illustration of symbols and abbreviations used this the methodology.

The research considered the following two hypotheses for inferential analysis,

1. Null hypothesis H_0_ (${µ}_{2-}{µ}_{1}=0$): There is no statistical significance of results between the proposed **QCM** algorithm and other existing algorithms (DnC, SLA, and Hy-IoT) for QoS-enabled layered-based clustering for reactive flooding in the Internet of Things.
2. Alternative hypothesis H_1_ (${µ}_{2-}{µ}_{1}>0$): There is statistical significant relationship between the proposed **QCM** algorithm and other existing algorithms (DnC, SLA, and Hy-IoT) for QoS-enabled layered-based clustering for reactive flooding in the Internet of Things.

Further, the researcher performed a T-test and an ANOVA test for the above hypothesis testing.

Let the Sample mean difference be

(1) $$\bar{d}={µ}_{2-}{µ}_{1}$$

where ${µ}_{1}$ is the sample mean of the data set of results for the first algorithm and ${µ}_{2}$ is the sample mean of the data set of results for the second comparable algorithm.

Sample standard deviation

(2) $$S_{D}=\sqrt{\frac{1}{N-1}\overset{N}{\underset{i=1}{\sum }}{\left(x_{i}-\bar{d}\right)}^{2}}$$

Here, data points are $x_{1\;,\;}x_{2,\;}x_{3,\;\;\;,\;\;\;\;,\;}x_{N\;}$ in the data sets of results of the two comparable algorithms.

Paired Sample T-test:

(3) $$T=\frac{\bar{d}-0}{S_{D}/\sqrt{n}}$$

Here, *n* represents the number of observations. We find the probability value (p) by observing the test statistics under the null and alternative hypothesis. This probability would help to identify the magnitude of the significance in the results for our proposed **QCM** algorithm.

The test calculates the probability value (P-value) based on the data sets of the results for different comparable algorithms. The standard confidence interval is 0.05 (95% confidence interval); P-values less than 0.05 are considered statistically significant. On the contrary, P-values larger than the chosen confidence interval infer that performances of comparable algorithms have no statistical significance and hence no algorithm outperformed the other algorithms in this comparison.

In addition, the authors performed an “ANOVA test” [35,36,37] to validate the performance measure of algorithms. The ANOVA test contains the following features,

Mean square for samples,

(4) $$MSR=\frac{SSR}{k-1}$$

Similarly, the mean square for error,

(5) $$MSE=\frac{SSE}{n-k}$$

Now the F statistics becomes

(6) $$F=\frac{MSR}{MSE}$$

This research, by statistical means, evaluates the performance of different QoS-enabled layered-based clustering algorithms for reactive flooding in the Internet of Things with the following measures.

1. **Inferential analysis in terms of Energy Consumption**
  - Energy consumption with different intervals of traffic
  - Energy consumption with malicious mote
  - Energy consumption with malicious mote with a realistic condition
2. **Inferential analysis in terms of Delay**
  - Delay with different intervals of traffic
  - Delay with malicious mote
  - Delay with malicious mote with a realistic condition
3. **Inferential analysis in terms of Throughput**
  - Throughput with different intervals of traffic
  - Throughput with malicious mote
  - Throughput with malicious mote with a realistic condition

Based on our hypothesis theories stated above, we find the probability value “P” employing the statistical t-test to figure out acceptance or rejection of our Null hypothesis (or alternative hypothesis) as a metric for performance evaluation of proposed and existing algorithms. 

## 5. Results

The performance estimation and evaluation of the proposed technique against up-to-date DnC, SLA [2,3] and Hy-IoT [32] methods for tracing and mitigating the unwanted and redundant reactive flooding are described in this section. Routing protocol and MDP protocol [24] are ad-hoc routing and Contiki, respectively. To obtain the appropriate results, simulation is performed 60 times based on the following three scenarios.

- Scenario based on varied intervals of traffic: This condition plays an important role to gauge and ensure the effectiveness of flooding attacks and to regulate the defensive techniques in varying intervals of traffic. The range for the traffic interval is set as (1 s to 10 s), where 1 s is faster and 10 s is slower.
- Scenario based on a varied number of mischievous motes: this condition is favorable in analyzing the impact of a flooding attack on the network and to take the appropriate action to counter mischievous motes. Motes (2,6,10,15) are set as mischievous motes, and the interval of traffic is set to (1 s) where 1 s is referred as the fastest traffic in the network.
- Condition based on realistic scenario: In this conditional scenario, motes are restricted to not transfer the route query information simultaneously; they are only allowed to transfer route query requests at different intervals of time. These intervals are randomly set from 1 s to 10 s.

Further, this section describes the inferential analysis of experimental results related to the performance evaluation and validation of the proposed **QCM (Query Control Mechanism)** algorithm (Khan, F. A., Noor, R. M., Mat Kiah, M. L., Noor, N. M., Altowaijri, S. M., & Rahman, A. U., 2019). We present here the rejection of the Null hypothesis and acceptance of the alternative hypothesis since the **QCM** algorithm outperforms (95% confidence interval) the existing algorithms, i.e., DnC, SLA, and Hy-IoT for QoS-enabled layered-based clustering for reactive flooding in the Internet of Things.

**Case 1: Inferential analysis in terms of energy consumption**

Here, in this case, we discuss the performance evaluation in terms of energy consumption with three different scenarios, i.e., different intervals of traffic, malicious mote and malicious mote with realistic conditions.

**Figure 3:**
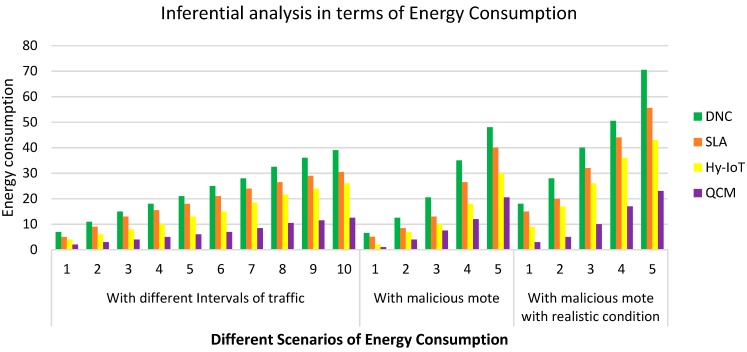
Energy Consumption with respect to different scenarios.

Figure 3 depicts energy consumptions with respect to different scenarios, i.e., different intervals of traffic, with malicious mote and with malicious mote and with realistic conditions. The proposed QCM technique outperformed DnC, SLA, and Hy-IoT approaches in term of dropping the average consumption of energy. Because the proposed technique is capable of detecting flooder motes and detaching them from the network, this reduces the level of energy consumption that arises during redundant and unwanted flooding attacks, whereas the average energy consumption of DnC and SLA is approximately 21 and 18%, respectively, from (1 to 5) seconds of intervals, and this ratio continuously rises as the interval increases. However, in the case of the proposed mechanism, the ratio of the consumption of energy falls to 6% as compared to 13% in the existing Hy-IoT approach.

We can observe that **QCM** achieves the lowest energy consumption as compared to other prevailing algorithms in the described scenarios.

Table 2 presents the statistical observations of data related to the inferential analysis of **QCM** and other existing algorithms. We can see that the statistical significant value **P** is less than our chosen confidence interval of 0.05 which is evidence that our proposed **QCM** algorithm outperforms the existing algorithms. Hence, the Null hypothesis is rejected and **QCM** achieves the significant prediction value in the desired confidence interval.

An exact realistic analysis of QCM is conducted to find the level of mischievous motes during flooding expansion in the network. It is evident from the result that in the presence of malicious motes, the level of energy consumption increases gradually. At malicious mote 2, the levels of energy consumption are approximately (8 and 5%) for DnC and SLA approaches respectively, and at malicious mote 15, this consumption level reaches approximately (48 and 40%). Hence, by introducing QCM, this level falls to approximately (2, 4, and 20%) at malicious mote (2, 6 and 15), respectively.

Table 3 presents the ANOVA test statistics of the proposed QCM algorithm compared with other algorithms. We can find here that “F statistics” values are sufficiently larger than “F critical values”. In addition, the “P values” are less than 0.05, which achieves our 95% confidence interval, showing that the proposed QCM algorithm outperforms the existing algorithms evaluated through inferential analysis.

**Case 2: Inferential analysis in terms of Delay**

The effect of traffic delay on the number of malicious motes, the time interval and malicious motes under realistic conditions are described in this section. QCM outperformed DnC, SLA and Hy-IoT by having the least traffic delays. QCM has the ability to detect, pause and detach the flooding mote from the network, which helped in improving its performance. On the other hand, the redundant and unwanted queries were also removed by detaching the flooding motes.

Here, in this case, we discuss the performance evaluation in terms of delay with three different scenarios, i.e., different intervals of traffic, malicious mote and malicious mote with realistic conditions.

**Figure 4:**
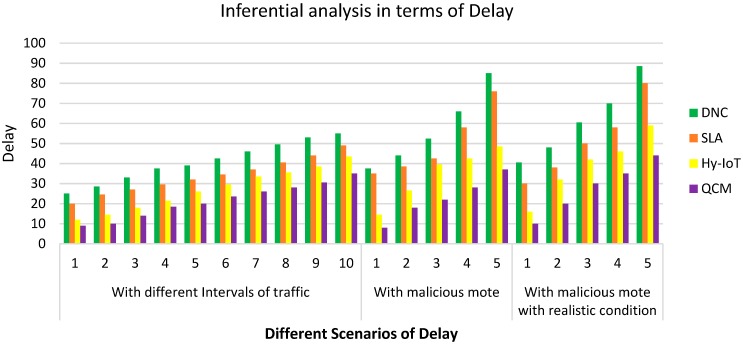
Delay with different intervals of traffic.

Figure 4 presents the “delay” with respect to different scenarios, i.e., different intervals of traffic, with malicious mote and with malicious mote and with realistic conditions. We can observe that **QCM** achieves the lowest delay as compared to other prevailing algorithms in described scenarios.

Table 4 presents the statistical observations of data related to the inferential analysis of **QCM** and other existing algorithms. We can see that the statistical significance value **P** is less than our chosen confidence interval 0.05 which is evidence that our proposed **QCM** algorithm outperforms the existing algorithms. Hence, the Null hypothesis is rejected and **QCM** achieves the significant prediction value in the desired confidence interval.

Table 5 presents the ANOVA test statistics of the proposed QCM algorithm compared with other algorithms. We can find here that “F statistics” values are sufficiently larger than “F critical values”. In addition, the “P values” are less than 0.05, which achieves our 95% confidence interval, showing that the proposed QCM algorithm outperforms the existing algorithms evaluated through inferential analysis.

**Case 3: Inferential analysis in terms of throughput**

The proposed QCM is compared with DnC, SLA, and Hy-IoT using network throughput which we refer to as QoS. Here, QoS is measured for these four flooding mechanisms using three scenarios: time interval, increasing number of malicious motes and malicious motes with realistic network conditions.

Here, in this case, we discuss the performance evaluation in terms of throughput with three different scenarios, i.e., different intervals of traffic, malicious mote and malicious mote with realistic conditions.

**Figure 5:**
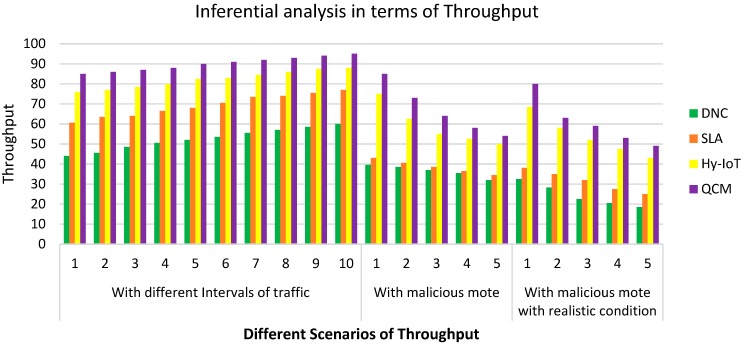
Throughput with different intervals of traffic.

Figure 5 presents the “Throughput” with respect to different scenarios, i.e., different intervals of traffic, with malicious mote and with malicious mote and with realistic conditions. We can observe that **QCM** achieves the highest throughput as compared to other prevailing algorithms in the described scenarios.

Table 6 presents the statistical observations of data related to the inferential analysis of **QCM** and other existing algorithms. We can see that the statistically significant value **P** is less than our chosen confidence interval of 0.05 which is evidence that our proposed **QCM** algorithm outperforms the existing algorithms. Hence, the Null hypothesis is rejected and **QCM** achieves the significant prediction value in the desired confidence interval.

Table 7 presents ANOVA test statistics of the proposed QCM algorithm compared with other algorithms. We can find here that “F statistics” values are sufficiently larger than “F critical values”. In addition, the “P values” are less than 0.05, which achieves our 95% confidence interval, showing that the proposed QCM algorithm outperforms the existing algorithms evaluated through inferential analysis.

## 6. Discussion (Hypothesis Testing)

This research was based on the hypothesis that the proposed **QCM (Query Control Mechanism)** algorithm (Khan, F. A., Noor, R. M., Mat Kiah, M. L., Noor, N. M., Altowaijri, S. M., & Rahman, A. U., 2019) outperforms the other existing algorithms, i.e., DnC, SLA, and Hy-IoT for QoS-enabled layered-based clustering for reactive flooding in the Internet of Things. The study elaborated numerous defensive techniques against unwanted and redundant routing queries which lead to heavy network traffic and flooding in IoT networks. In this study, the authors implemented the reactive part Interlayer clustering (IELC) of Cluster based flooding (CBF) and proposed a Query control mechanism (QCM) to detect and terminate the unwanted and redundant queries based on link signal strength, consistency of query packet and query limit threshold.

It is evident from the results that the proposed QCM had superior performance compared with the state of the art defensive techniques in terms of the average consumption of energy, traffic delay, and QoS which we referred to as network throughput. Thus, QCM drops the average consumption of energy to a significant rate as compared to the DnC, SLA, and Hy-IoT under varying intervals of traffic. The performance of QCM is also better regarding average consumption of energy with malicious motes against the traditional approaches by droping the consumption at different motes. Additionally, QCM also exhibits dominant performance regarding network delay by decreasing the delay as compared to the state of the art.

In the case of malicious motes, the proposed QCM drops the network delay to a significant level. Lastly, QCM enhances the amount of QoS to a greater extent as compared to Hy-IoT. The Proposed QCM technique employs the Query Limit Threshold (QLT) for detecting and terminating the redundant and unwanted query request packets, and in this way boosts the IoT network performance in terms of signal strength of query packets and improves the location consistency checking of connected motes to keep the network away from reactive flooding attacks. 

This performance clearly shows the difference between our approach and the contemporary approaches. We plan to extend this work in the future by considering a discrete component circuit implementation model using Bouali’s system to detect some other attacks in IoT by extending the number and types of motes in order to test the reliability of our approach in the presence of many motes. Also, we plan to include the proactive part Intralayer clustering (IALC) of the CBF, which is favorable in high priority and less delay IoT networks, i.e., smart transportation, smart health, and smart security, and to model a physical prototype for it.

The statistical tests calculated the probability value, “P-value”, based on the data sets of results for different comparable algorithms. We kept the standard confidence interval as 0.05 to determine the 95% confidence interval. “P-values” less than 0.05 were considered statistically significant. On the contrary, “P-values” larger than the chosen confidence interval inferred that the performance of comparable algorithms had no statistical significance for results and hence no algorithm outperformed the other algorithms in this comparison.

This research employed statistical measures to evaluate the performance of different QoS-enabled layered-based clustering algorithms for reactive flooding in the Internet of Things with the following measures. The inferential analysis was performed in the context of **Energy Consumption** (with different intervals of traffic, with malicious mote and with malicious mote with realistic conditions). Similarly, Inferential analysis was performed in terms of **Delay** (with different intervals of traffic, with malicious mote and with malicious mote with realistic conditions). Further, the research estimated the inferential measures in the context of **Throughput** (with different intervals of traffic, with malicious mote and with malicious mote with realistic conditions).

Based on our hypothesis theories stated earlier, we found the probability value “P” (in all statistical evaluations) remained less than 0.05, which rejected the Null hypothesis that there was no statistical significance of results for the **proposed QCM algorithm** as compared to the results of other existing algorithms, i.e., DnC, SLA, and Hy-IoT for QoS-enabled layered-based clustering for reactive flooding in the Internet of Things. Further, in the context of the alternative hypothesis, the evaluation of performance measures revealed that the alternative hypothesis was accepted since the **proposed QCM algorithm** outperformed the other existing algorithms.

## 7. Conclusions

This research article presented a statistical performance evaluation of different query control mechanisms. The performances of such query control mechanisms rely on minimizing the energy consumption, cost and network flooding. This article simulated and evaluated the performance measure of different query control mechanisms for QoS-enabled layered-based clustering for reactive flooding in the Internet of Things. By statistical means, we infer the significant achievement of the QCM algorithm (Khan, F. A., Noor, R. M., Mat Kiah, M. L., Noor, N. M., Altowaijri, S. M., & Rahman, A. U., 2019) that outperformed the prevailing algorithms, i.e., DnC, SLA, and Hy-IoT for identification and elimination of redundant flooding queries. The inferential analysis for performance evaluation of algorithms was measured in terms of energy consumption with energy consumption, delay and throughput with different intervals of traffic, malicious mote and malicious mote with realistic conditions. It is evident from the results that the QCM algorithm outperforms the existing algorithms, depicting the statistical probability value “P” < 0.05, indicating the performance of QCM significantly achieved the 95% confidence interval. Hence, the performance of the QCM algorithm is significant as compared to the performance of other algorithms. 

## Figures and Tables

**Table 1:** Illustration of symbols and notations used in the manuscript.

Symbol or Notation	Meaning
H_0_	Null Hypothesis
H_1_	Alternative Hypothesis
µ	Mean of sample values
DnC	Divide-and-Conquer method
SLA	Service-Level Agreements
Hy-IoT	Hybrid energy aware clustered protocol for IoT heterogeneous network
QoS	Quality of Service
S_D_	Standard Deviation
∑	Summation of a data series
MSR	Mean squares for samples
MSE	Mean squares for errors
SSR	Sum of squares for samples
SSE	Sum of squares for errors
QCM	Query control mechanism
P	Probability

**Table 2:** Inferential analysis of the proposed **QCM** algorithm in terms of energy consumption scenarios.

**“Energy consumption” with different intervals of traffic**			
***Statistics***	***QCM and DNC***	***QCM and SLA***	***QCM and Hy-IoT***
Pearson Correlation	0.995898024	0.985945791	0.998690321
t Stat	−7.234140089	−7.658424991	−5.902772999
**P (T ≤ t) one-tail**	**0.000024**	**0.000016**	**0.000114**
t Critical one-tail	1.833112933	1.833112933	1.833112933
**P (T ≤ t) two-tail**	**0.000049**	**0.000031**	**0.000228**
t Critical two-tail	2.262157163	2.262157163	2.262157163
**“Energy consumption” with malicious mote**			
***Statistics***	***QCM and DNC***	***QCM and SLA***	***QCM and Hy-IoT***
Pearson Correlation	0.991199989	0.990539256	0.997641141
t Stat	−3.69153586	−3.080170745	−2.910781287
**P (T ≤ t) one-tail**	**0.010495227**	**0.018462731**	**0.021822006**
t Critical one-tail	2.131846786	2.131846786	2.131846786
**P (T ≤ t) two-tail**	**0.020990453**	**0.036925463**	**0.043644012**
t Critical two-tail	2.776445105	2.776445105	2.776445105
**“Energy consumption” with malicious mote with realistic conditions**			
***Statistics***	***QCM and DNC***	***QCM and SLA***	***QCM and Hy-IoT***
Pearson Correlation	0.988654058	0.997737729	0.985054247
t Stat	−5.47596162	−5.744022068	−5.700342309
**P (T ≤ t) one-tail**	**0.002706491**	**0.002276306**	**0.002340359**
t Critical one-tail	2.131846786	2.131846786	2.131846786
**P (T ≤ t) two-tail**	**0.005412981**	**0.004552613**	**0.004680719**
t Critical two-tail	2.776445105	2.776445105	2.776445105

**Table 3:** ANOVA statistics of the proposed **QCM** algorithm in terms of energy consumption scenarios.

**“Energy consumption” with different intervals of traffic**						
***Source of Variation***	***SS***	***df***	***MS***	***F***	***P-value***	***F crit***
Between Groups	1454.17075	3	484.7235833	7.408406	0.000547983	2.866265551
Within Groups	2355.439	36	65.42886111			
Total	3809.60975	39				
**“Energy consumption” with malicious mote**						
***Source of Variation***	***SS***	***df***	***MS***	***F***	***P-value***	***F crit***
Between Groups	673.1095	3	224.3698333	4.334246	0.029824869	3.238871517
Within Groups	2690.596	16	168.16225			
Total	3363.7055	19				
**“Energy consumption” with malicious mote with realistic conditions**						
***Source of Variation***	***SS***	***df***	***MS***	***F***	***P-value***	***F crit***
Between Groups	2399.974	3	799.9913333	3.349041	0.04550264	3.238871517
Within Groups	3821.948	16	238.87175			
Total	6221.922	19				

**Table 4:** Inferential analysis of the proposed **QCM** algorithm in terms of “delay” scenarios.

**“Delay” with different intervals of traffic**			
***Statistics***	***QCM and DNC***	***QCM and SLA***	***QCM and Hy-IoT***
Pearson Correlation	0.994443621	0.988982601	0.996492201
t Stat	−35.04330697	−28.82155963	−8.856366815
**P (T ≤ t) one-tail**	**0.000000**	**0.000000**	**0.000005**
t Critical one-tail	1.833112933	1.833112933	1.833112933
**P (T ≤ t) two-tail**	**0.000000**	**0.000000**	**0.000010**
t Critical two-tail	2.262157163	2.262157163	2.262157163
**“Delay” with malicious mote**			
***Statistics***	***QCM and DNC***	***QCM and SLA***	***QCM and Hy-IoT***
Pearson Correlation	0.971603496	0.941343288	0.960608021
t Stat	−8.753903055	−7.964645156	−5.894374846
**P (T ≤ t) one-tail**	**0.000469295**	**0.000673192**	**0.002071644**
t Critical one-tail	2.131846786	2.131846786	2.131846786
**P (T ≤ t) two-tail**	**0.00093859**	**0.001346383**	**0.004143289**
t Critical two-tail	2.776445105	2.776445105	2.776445105
**“Delay” with malicious mote with realistic conditions**			
***Statistics***	***QCM and DNC***	***QCM and SLA***	***QCM and Hy-IoT***
Pearson Correlation	0.978790154	0.971228078	0.994541157
t Stat	−11.51506032	−7.200852222	−7.656162383
**P (T ≤ t) one-tail**	**0.000162379**	**0.00098563**	**0.000782041**
t Critical one-tail	2.131846786	2.131846786	2.131846786
**P (T ≤ t) two-tail**	**0.000324759**	**0.001971259**	**0.001564082**
t Critical two-tail	2.776445105	2.776445105	2.776445105

**Table 5:** ANOVA statistics of the proposed **QCM** algorithm in terms of “Delay” scenarios.

**“Delay” with different intervals of traffic**						
***Source of Variation***	***SS***	***df***	***MS***	***F***	***P-value***	***F crit***
Between Groups	1454.171	3	484.7236	7.408406	0.000548	2.866266
Within Groups	2355.439	36	65.42886			
Total	3809.61	39				
**“Delay” with malicious mote**						
***Source of Variation***	***SS***	***df***	***MS***	***F***	***P-value***	***F crit***
Between Groups	673.1095	3	224.3698	4.334246	0.029825	3.238872
Within Groups	2690.596	16	168.1623			
Total	3363.706	19				
**“Delay” with malicious mote with realistic conditions**						
***Source of Variation***	***SS***	***df***	***MS***	***F***	***P-value***	***F crit***
Between Groups	2399.974	3	799.9913	3.349041	0.045503	3.238872
Within Groups	3821.948	16	238.8718			
Total	6221.922	19				

**Table 6:** Inferential analysis of the proposed **QCM** algorithm in terms of “Throughput” scenarios.

**“Throughput” with different intervals of traffic**			
***Statistics***	***QCM and DNC***	***QCM and SLA***	***QCM and Hy-IoT***
Pearson Correlation	0.993765698	0.992949209	0.997823573
t Stat	59.53356302	29.60983067	28.80340889
**P (T ≤ t) one-tail**	**0.000000**	**0.000000**	**0.000000**
t Critical one-tail	1.833112933	1.833112933	1.833112933
**P (T ≤ t) two-tail**	**0.000000**	**0.000000**	**0.000000**
t Critical two-tail	2.262157163	2.262157163	2.262157163
**“Throughput” with malicious mote**			
***Statistics***	***QCM and DNC***	***QCM and SLA***	***QCM and Hy-IoT***
Pearson Correlation	0.903419023	0.986206076	0.988735878
t Stat	6.867764974	6.871919521	6.044877215
**P (T ≤ t) one-tail**	**0.001177169**	**0.00117451**	**0.001888935**
t Critical one-tail	2.131846786	2.131846786	2.131846786
**P (T ≤ t) two-tail**	**0.002354338**	**0.002349021**	**0.003777869**
t Critical two-tail	2.776445105	2.776445105	2.776445105
**“Throughput” with malicious mote with realistic conditions**			
***Statistics***	***QCM and DNC***	***QCM and SLA***	***QCM and Hy-IoT***
Pearson Correlation	0.960853622	0.938109794	0.989516045
t Stat	12.24631557	9.025002168	5.969620058
**P (T ≤ t) one-tail**	**0.000127655**	**0.000417442**	**0.001977709**
t Critical one-tail	2.131846786	2.131846786	2.131846786
**P (T ≤ t) two-tail**	**0.000255309**	**0.000834884**	**0.003955418**
t Critical two-tail	2.776445105	2.776445105	2.776445105

**Table 7:** ANOVA statistics of the proposed **QCM** algorithm in terms of “Throughput” scenarios.

**“Throughput” with different intervals of traffic**						
***Source of Variation***	***SS***	***df***	***MS***	***F***	***P-value***	***F crit***
Between Groups	1454.17075	3	484.7236	7.408406	0.000548	2.866266
Within Groups	2355.439	36	65.42886			
Total	3809.60975	39				
**“Throughput” with malicious mote**						
***Source of Variation***	***SS***	***df***	***MS***	***F***	***P-value***	***F crit***
Between Groups	673.1095	3	224.3698	4.334246	0.029825	3.238872
Within Groups	2690.596	16	168.1623			
Total	3363.7055	19				
**“Throughput” with malicious mote with realistic conditions**						
***Source of Variation***	***SS***	***df***	***MS***	***F***	***P-value***	***F crit***
Between Groups	2399.974	3	799.9913	3.349041	0.045503	3.238872
Within Groups	3821.948	16	238.8718			
Total	6221.922	19				
